# Phytohormone pathways as targets of pathogens to facilitate infection

**DOI:** 10.1007/s11103-016-0452-0

**Published:** 2016-02-15

**Authors:** Ka-Wai Ma, Wenbo Ma

**Affiliations:** Department of Plant Pathology and Microbiology, University of California, Riverside, CA 92521 USA; Center for Plant Cell Biology, University of California, Riverside, CA 92521 USA

**Keywords:** Plant immunity, Pathogen effectors, Phytohormones, Pathogenicity, Virulence, Bacterial toxins

## Abstract

Plants are constantly threatened by potential pathogens. In order to optimize the output of defense against pathogens with distinct lifestyles, plants depend on hormonal networks to fine-tune specific responses and regulate growth-defense tradeoffs. To counteract, pathogens have evolved various strategies to disturb hormonal homeostasis and facilitate infection. Many pathogens synthesize plant hormones; more importantly, toxins and effectors are produced to manipulate hormonal crosstalk. Accumulating evidence has shown that pathogens exert extensive effects on plant hormone pathways not only to defeat immunity, but also modify habitat structure, optimize nutrient acquisition, and facilitate pathogen dissemination. In this review, we summarize mechanisms by which a wide array of pathogens gain benefits from manipulating plant hormone pathways.

## Introduction

In order to complete an infection cycle, phytopathogens need to enter plant tissues through physical barriers, overcome defense responses mounted by the plant immune system, obtain nutrients for proliferation, and eventually be disseminated to a new host. During the co-evolutionary arms race with plants, successful pathogens evolved virulence factors such as toxins and secreted proteins (aka effectors) to modulate plant physiology (Bender et al. 1999; Torto-Alalibo et al. 2009; Dou and Zhou 2012; Dangl et al. 2013). A prominent and extensively studied example is the type III secreted effectors, which are injected by Gram negative bacterial pathogens directly into plant cells (Deslandes and Rivas 2012; Feng and Zhou 2012). Eukaryotic filamentous pathogens including fungi and oomycetes also produce a large number of effectors that can function inside host cells (Torto-Alalibo et al. 2009; Wawra et al. 2012; Giraldo and Valent 2013). Over the past decade, substantial efforts have been invested to understand how effectors facilitate pathogen colonization and disease development. Through these studies, phytohormone pathways have emerged as important virulence targets.

Plant hormones are small molecules that affect a broad range of processes during growth and stress responses (Depuydt and Hardtke 2011; Pieterse et al. 2012; Vanstraelen and Benkova 2012). By manipulating plant hormonal pathways, pathogens can further benefit through two mechanisms: one, they can suppress defense responses regulated by the “stress” hormones in order to accomplish colonization in plant tissues; two, they can hijack plant development and nutrient allocation processes regulated by the “growth” hormones to facilitate sustained colonization and dissemination.

In this review, we classify major plant hormones into “stress” and “growth” hormones and discuss the mechanisms by which microbial pathogens interfere with their accumulation and/or signaling. Due to space constrains, we will focus on pathogen effectors and toxins that have been demonstrated to directly manipulate hormonal networks in plants as virulence strategies to increase pathogen fitness. Examples from a wide variety of pathogens (viruses, bacteria, fungi, oomycetes and herbivores) using different infection strategies will be discussed.

## Plant hormones as key regulators of immunity

Unlike animals that can move and adapt to survive suboptimal conditions, plants are sessile; therefore, the ability to defeat pathogen infection is critical to survival. Threatened by a large variety of pests and pathogens, plants have evolved a sophisticated innate immune system (Spoel and Dong 2012). A basal tier of plant immunity is activated by conserved molecular signatures called pathogen/microbe-associated molecular patterns (PAMPs/MAMPs), which can be recognized by receptor-like kinases known as pattern recognition receptors (PRRs) (Zipfel 2014). Pattern-triggered immunity (PTI) is associated with a series of physiological responses that confer effective, broad-spectrum defense against the majority of potential pathogens (Bigeard et al. 2015). Shortly after pathogen perception, extensive transcription reprogramming of genes involved in hormonal signaling occurs (De Vos et al. 2005), suggesting a key regulatory role of hormones in mounting defense responses.

**Fig. 1:**
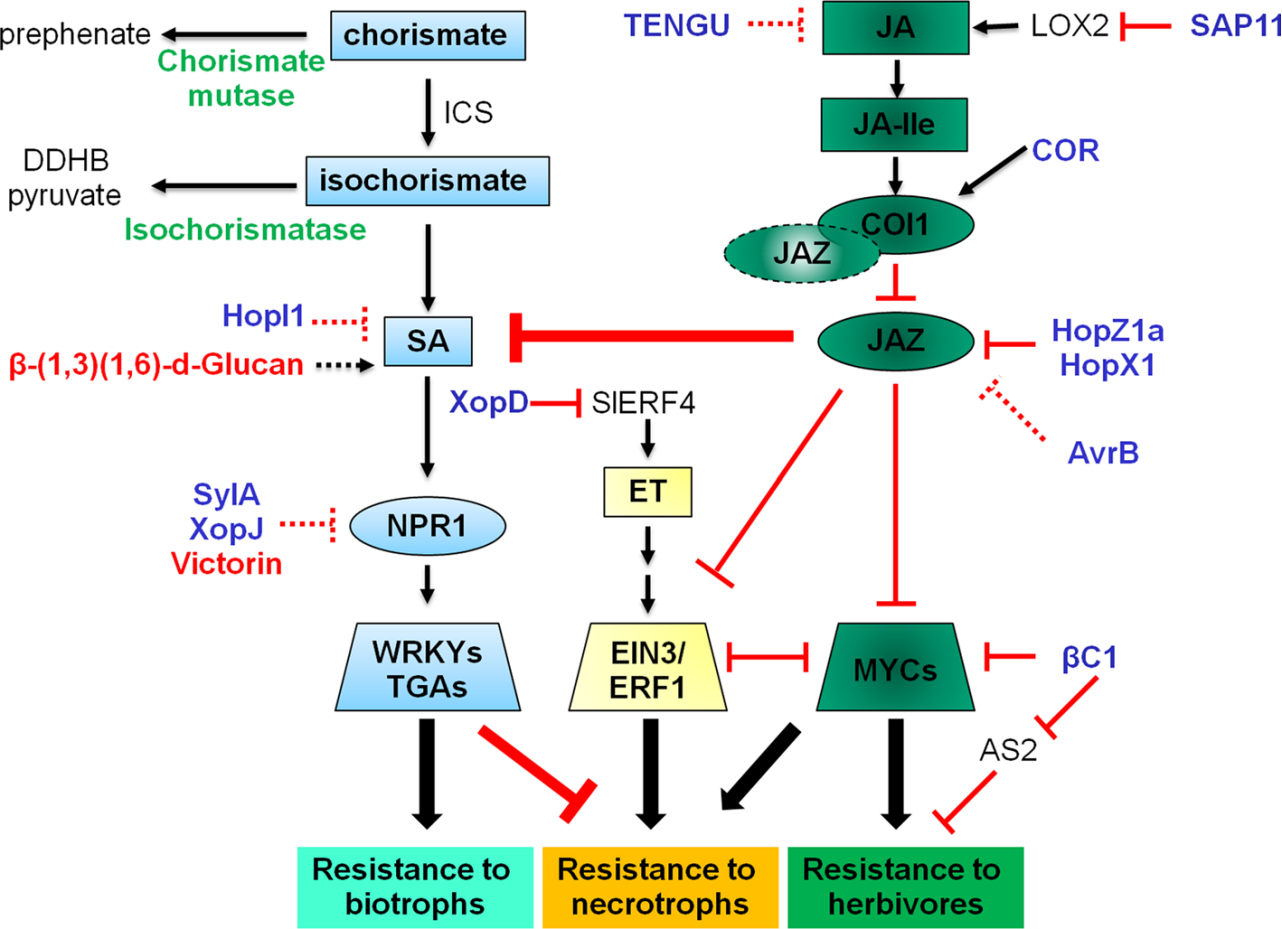
A diagram showing the crosstalk among SA, JA and ET signaling pathways and their roles in defense against pathogens/herbivores using distinctive infection strategies. Effectors and toxins target SA, JA and ET signaling to suppress plant defense are presented. Virulence factors produced by biotrophs/hemibiotrophs are highlighted in *blue*, and those produced by necrotrophs are highlighted in *red*. Chorismate mutase and isochromatases are produced by both biotrophs and necrotrophs and are highlighted in *green*. *Broken lines* indicate indirect manipulation processes or unknown mechanisms

Major plant hormones that regulate defense responses include salicylic acid (SA), jasmonic acid (JA) and ethylene (ET). Generally speaking, SA plays a key role in defense against pathogens feeding on live tissues, i.e. with a biotrophic lifestyle; and JA/ET is critical to defense against pathogens feeding on dead tissues, i.e. with a necrotrophic lifestyle (Glazebrook 2005). In addition, JA alone is prominent in defense against herbivores (Fig. 1). An important concept that has been established over the years is the antagonism between SA and JA/ET pathways in response to pathogens with a specific lifestyle (Spoel and Dong 2008; Van der Does et al. 2013). However, analysis using a mutant that is defective in SA, JA and ET pathways supported a more synergistic view in that all three hormones contribute positively to defense against various pathogens with one hormone sector makes larger contributions than others in response to a specific infection style (Tsuda et al. 2009).

### Toxins and effectors targeting salicylic acid accumulation

SA is the first plant hormone with a demonstrated role in defense (White 1979) and has since been studied extensively. Provided the importance of SA in plant defense, many pathogens have evolved strategies to target the SA pathway, either at the level of biosynthesis/accumulation or downstream signaling (Fig. 1).

A main virulence strategy is to suppress SA accumulation through the activity of secreted enzymes that metabolize SA precursors. For example, the biotrophic fungal pathogen *Ustilago maydis*, which causes maize smut, produces a chorismate mutase (Cmu1) during infection. Following pathogen perception, SA is produced from chorismate via the intermediate isochorismate through the activity of the isochorismate synthase (ICS) (Wildermuth et al. 2001). Cmu1 converts chorismate to prephenate, potentially lowering the availability of plastid chorismate for SA synthesis (Djamei et al. 2011). Indeed, maize infected with a *Cmu1* mutant of *U. maydis* accumulated more SA and exhibited attenuated disease symptoms compared with plants infected with wild-type strain (Djamei et al. 2011). Many biotrophic and hemibiotrophic pathogens produce chorismate mutases that can potentially benefit infection (Djamei et al. 2011). Interestingly, the necrotrophic fungal pathogen *Sclerotinia sclerotiorum* also produces a putative chorismate mutase. Pretreatment of the SA analog benzothiadiazole (BTH) on rapeseed plants resulted in an approximately 40 % reduction on lesion sizes caused by *S. sclerotiorum*, suggesting a positive role of SA in defense against *S. sclerotiorum* (Novakova et al. 2014). This observation indicates that the putative chorismate mutase produced by *S. sclerotiorum* may promote infection by reducing SA accumulation.

Another secreted enzyme with the ability to suppress SA accumulation is isochorismatase, which is produced by the hemibiotrophic oomycete pathogen *Phytophthora sojae* and the necrotrophic fungal pathogen *Verticillium dahlia*. Isochorismatase hydrolyzes isochorismate to 2,3-dihydro-2,3-dihydroxybenzoate (DDHB) and pyruvate. As such, *P. sojae* and *V. dahlia* reduce the SA levels in their specific hosts to facilitate infection (Liu et al. 2014). These studies demonstrate the suppression of SA accumulation as a common virulence strategy employed by both biotrophic and necrotrophic pathogens.

Enhanced SA accumulation may benefit pathogens using a distinctive infection strategy and in specific host-pathogen interactions. For instance, the broad host-range, necrotrophic, fungal pathogen *Botrytis cinerea* produces exopolysaccharide *β*-(1,3)(1,6)-d-glucan, which acts as an essential virulence factor by inducing SA accumulation, and hence suppressing JA signaling, in tomato (El Oirdi et al. 2011). Other interesting observations are that the mucus secreted by molluscan slugs and snails, and the honeydew deposited by whitefly could induce the expression of the SA marker gene *PR1* (Meldau et al. 2014; VanDoorn et al. 2015). Intriguingly, SA was found in the mucus of the slug *Deroceras reticulatum* (Kastner et al. 2014) and honeydew of the sweetpotato whitefly *Bemisia tabaci* (VanDoorn et al. 2015). Since the activation of SA signaling may suppress JA-mediated defense against herbivores, it would be interesting to investigate whether these herbivores-produced SA actually play a role in promoting infestation.

In addition to the above virulence factors with relatively clear SA manipulation mechanisms, other effectors have also been suggested to affect SA accumulation. For example, HopI1 produced by the hemibiotrophic bacterial pathogen *Pseudomonas syringae* suppresses SA accumulation in chloroplasts. HopI1 directly interacts with the heat shock protein Hsp70 and alters chloroplast thylakoid structure (Jelenska et al. 2007, 2010). However, how HopI1 interferes with SA production remains elusive.

### Toxins and effectors targeting salicylic acid signaling

NPR1 (NONEXPRESSOR OF PR GENES 1) is an essential component in SA-dependent defense. Upon pathogen perception, SA potentiates the reduction of the oligomeric form of NPR1 in cytoplasm to a monomeric form through redox changes (Mou et al. 2003). Monomeric NPR1 is then relocated to the nucleus and activates the expression of SA-responsive genes. NPR1-regulated SA signaling is tightly regulated through post-translational modifications and proteasomal degradation. In particular, proper turnover of NPR1 is required for the perpetuation of SA response (Tada et al. 2008; Spoel et al. 2009). Therefore, it is not surprised that NPR1 turnover is manipulated by various pathogens for the benefit of disease development.

The bacterial pathogen *P. syringae* produces a peptide toxin, syringolin A (SylA), which acts as a potent inhibitor of proteasomes (Groll et al. 2008; Schellenberg et al. 2010). SylA can diffuse through plant vasculature, generating a gradient of SA-insensitive cells and suppressing SA-mediated defense both at the initial infection site and in surrounding tissues (Misas-Villamil et al. 2013). It is postulated that SylA blocks the proteasomal degradation of NPR1 to interfere with SA signaling. The type III-secreted effector XopJ from the bacterial pathogen *Xanthomonas campestris* acts as a protease and degrades the 19S proteasome regulatory subunit REGULATORY PARTICLE AAA-ATPASE6 (RPT6). As such, XopJ inhibits NPR1 degradation and compromises anti-bacterial immunity in pepper (Üstün et al. 2013; Ustun and Bornke 2015).

The toxin victorin produced by the necrotrophic fungal pathogen *Cochliobolus victoriae* can suppress the activity of TRX-h5, a thioredoxin that regulates the redox status in plants (Sweat and Wolpert 2007). By inhibiting the redox reduction of NPR1, victorin may suppress SA-mediated defense via interfering with NPR1 relocation from cytosol to the nucleus. However, *C. victoriae* does not seem to take advantage of NPR1 manipulation by victorin as a virulence strategy. Rather, as a necrotrophic pathogen, *C. victoriae* uses victorin to induce plant cell death by hijacking the hypersensitive response triggered by LOV1, a resistance (R) protein that is activated when TRX-h5 is disturbed (Sweat and Wolpert 2007; Lorang et al. 2012). Indeed, *C. victoriae* only causes disease on Arabidopsis ecotypes that carry LOV1 (Lorang et al. 2004). Nonetheless, victorin represents a novel strategy of pathogen manipulation of NPR1.

### Toxins and effectors targeting jasmonate signaling

JA is the major defense hormone against necrotrophs and herbivores. Although the ability to suppress JA signaling has been implicated in some herbivores (Zarate et al. 2007; Bruessow et al. 2010; Glas et al. 2014), the responsible molecules or underlying mechanisms are unknown. On the contrary, many virulence factors produced by biotrophic or hemibitrophic pathogens take advantage of the antagonism between JA and SA pathways and activate JA signaling to promote infection (Fig. 1).

Jasmonic acid signaling is repressed by a group of proteins collectively known as the JASMONATE ZIM-DOMAIN proteins (JAZs) (Staswick 2008). Inside the nucleus, JAZs directly associates with the JA-responsive transcription factors and repress their functions (Pauwels et al. 2010; Shyu et al. 2012). Upon activation by pathogen perception or tissue damage, JA is synthesized from linolenic acid via 12-oxo-phytodienoic acid (OPDA) as the intermediate in plastids and conjugated with isoleucine to generate the bioactive form JA-Ile (Staswick and Tiryaki 2004; Fonseca et al. 2009). High levels of JA-Ile promote the formation of a receptor complex consisting of JAZs, the F-box protein COI1, and inositol pentakisphosphate. Association with COI1 leads to proteasomal degradation of JAZs, thereby de-repressing JA signaling (Thines et al. 2007; Melotto et al. 2008; Sheard et al. 2010).

The toxin coronatine (COR) produced by the bacterial pathogen *P. syringae* pv. *tomato* strain DC3000 (*Pto*DC3000) is by far the best studied example of virulence factors that can manipulate the JA pathway. Structurally mimicking JA-Ile, COR is ~1000 fold more effective in inducing the degradation of JAZs and acts as a robust inducer of JA signaling (Katsir et al. 2008). Plants respond to bacterial invasion by closing stomata in order to inhibit pathogen entry into the apoplastic space. Activation of JA signaling by COR promotes the entry of *Pto*DC3000 into leaf tissues by re-opening the closed stomata (Melotto et al. 2006). In addition, COR inhibits SA accumulation in plant cells, likely also through its activation of JA signaling (Zheng et al. 2012).

A COR-like compound, coronafacic acid (CFA)-L-Ile, is produced by various pathogenic *Streptomyces* species that cause potato scab disease (Bignell et al. 2010). CFA-L-Ile is required for the full virulence of *Streptomyces*; furthermore, application of CFA-L-Ile can induce hypertrophic outgrowths on potato, suggesting that this toxin may contribute to disease symptom development (Fyans et al. 2015). Although COR can also induce a similar phenotype on potato, whether the virulence function of CFA-L-Ile during *Streptomyces* infection is achieved through JA mimicking is unknown.

Acting as effective virulence factors, COR-like toxins are only produced by a small number of bacterial pathogens (for example, most *P. syringae* isolates do not produce COR) (Volksch and Weingart 1998; Hwang et al. 2005); therefore, it is not surprising to find additional pathogen strategies for JA manipulation. In particular, several type III effectors from *P. syringae* have recently been shown to directly or indirectly promote JAZ degradation. HopZ1a possesses an acetyltransferase activity and directly interacts with multiple JAZs in Arabidopsis and soybean. Acetylation of JAZs by HopZ1a promotes their degradation in a COI1-dependent manner and activates JA signaling (Jiang et al. 2013). Another *P. syringae* effector that activates JA signaling is HopX1, which acts as a cysteine protease and directly hydrolyzes JAZs in Arabidopsis (Gimenez-Ibanez et al. 2014). Recently, a third type III effector AvrB was shown to induce JAZs degradation, but through an indirect mechanism (Zhou et al. 2015). Similar to COR, HopZ1a and AvrB are also able to inhibit stomatal defense and promote bacterial entry to apoplastic space (Ma et al. 2015; Zhou et al. 2015). The findings that multiple virulence factors, including both toxins and effectors, manipulate the same host targets highlight the importance of JA pathway as a virulence target.

### Manipulation of jasmonate signaling to promote pathogen dissemination

Many bacterial and viral pathogens depend on insect vectors for transmission. Since JA is a major defense hormone against insects, these pathogens have evolved strategies to suppress JA accumulation and/or signaling in order to promote their dissemination.

Phytoplasmas are bacterial pathogens that depend on phloem-feeding leafhoppers for transmission. The Aster Yellows phytoplasma strain Witches’ Broom (AY-WB) produces a Sec-secreted effector called SAP11, which destabilizes the TEOSINTE BRANCHED1, CYCLOIDEA, PROLIFERATING CELL FACTORS (TCP) class of transcription factors in Arabidopsis (Ikeda and Ohme-Takagi 2014). Among them, TCP4 activates the expression of JA biosynthetic gene *LIPOXYGENASE 2* (*LOX2*) (Schommer et al. 2008). AY-WB infection or *SAP11* expression in Arabidopsis led to reduced *LOX2* expression and JA levels; as a result, the fecundity of leafhoppers was increased and the transmission of AY-WB was enhanced (Sugio et al. 2011). TENGU is another phytoplasma Sec-secreted effector that has been shown to suppress JA accumulation. TENGU represses the expression of two auxin-response factors ARF6 and ARF8 (Hoshi et al. 2009), which positively regulate JA biosynthesis (Reeves et al. 2012). It has been postulated that, similar to SAP11, TENGU may also facilitate leafhopper feeding, and hence phytoplasma transmission, by manipulating the JA pathway (Minato et al. 2014).

A similar scenario exists in the interaction between tomato yellow leaf curl China virus (TYLCCNV) and its whitefly vector. TYLCCNV produces βC1 as a virulence factor to suppress anti-herbivore volatile emission and promote whitefly survival (Jiu et al. 2007; Zhang et al. 2012). βC1 acts as a mimic of the plant regulator ASYMMETRIC LEAVES 2 (AS2) (Yang et al. 2008), which inhibits JA signaling (Nurmberg et al. 2007). In addition, βC1 also inhibits the dimerization of MYC2, a JA-responsive transcription factor required for the activation of the terpene synthetic gene *TERPENE SYNTHASE 10 (TPS10)* (Li et al. 2014). Interestingly, AS2 and MYC2 seem to regulate distinct subsets of JA-responsive genes (Li et al. 2014). As such, βC1 suppresses JA signaling to favor viral transmission by simultaneously manipulating two targets that regulate the JA response (Jiu et al. 2007; Zhang et al. 2012).

### Manipulation of ethylene levels by pathogens to promote infection

Together with JA, ethylene (ET) has been shown to regulate defense against necrotrophs (Thomma et al. 1999). As a gaseous hormone, ET is widely produced by many microorganisms including plant pathogens. For example, the bacterial pathogen *P. syringae* and *Ralstonia solanacearum*, as well as the fungal pathogen *B. cinerea*, can all produce ET (Weingart and Volksch 1997; Cristescu et al. 2002; Valls et al. 2006). Furthermore, the ability to produce ET is correlated with the full virulence of *P. syringae* strains on soybean and common bean (Weingart et al. 2001), indicating a potential virulence function of ET production in these pathogens.

An effector that can manipulate the ET pathway has been identified from the bacterial pathogen *Xanthomonas euvesicatoria.* XopD targets the tomato transcription factor SlERF4, which is involved in ET synthesis. Acting as a SUMO protease, XopD desumoylates SlERF4, promoting its degradation through the 26S proteasome. Consistently, silencing of *SIERF4* led to reduced ET accumulation and enhanced susceptibility of tomato to *X. euvesicatoria* (Kim et al. 2013). In addition, XopD delays chlorosis development on tomato leaves infected with *X. euvesicatoria,* which is likely due to its inhibitory effect on ET production (Kim et al. 2008, 2013). It is postulated that the modulation of ET-regulated senescence benefits bacterial proliferation by extending the infection period.

### Manipulation of the abscisic acid pathway by pathogens

More recently, abscisic acid (ABA), which has been extensively studied in plant response to abiotic stresses, is also implicated in defense responses. ABA acts as a negative regulator of defense against biotrophs (Cao et al. 2011), possibly due to its antagonistic effect on SA signaling (Audenaert et al. 2002; Xu et al. 2013). For example, exogenous application of ABA in rice significantly reduced the expression of two key regulators of SA-dependent defense signaling, *WRKY45* and *OsNPR1*, leading to hypersusceptibility to fungal infection (Jiang et al. 2010).

A couple of type III effectors produced by the bacterial pathogen *P. syringae* have been suggested to activate the ABA pathway during infection. AvrPtoB induces the expression of the ABA biosynthetic gene *9*-*CIS*-*EPOXYCAROTENOID DIOXYGENASE 3* (*NCED3*) and enhances ABA accumulation in Arabidopsis (de Torres-Zabala et al. 2007). Since AvrPtoB has been extensively characterized as a kinase inhibitor and directly targets receptor kinases (Shan et al. 2008; Cheng et al. 2011), its effect on ABA synthesis is likely indirect. Another type III effector HopAM1 also affects ABA signaling. Arabidopsis expressing HopAM1 exhibits increased sensitivity to ABA and enhanced susceptibility to bacterial infection (Goel et al. 2008). Although the correlation between these two observed phenotypes remains unclear, it is noteworthy that the hypersensitive response is associated with inhibited vascular water movement into the infection site. A drop in water potential presumably deprives the water supply that is required for bacteria to proliferate (Wright and Beattie 2004; Freeman and Beattie 2009). Interestingly, the virulence effect of HopAM1 was more prominent when the host plants were under water stress (Goel et al. 2008). It is intriguing to postulate that HopAM1 may create a microenvironment with higher water availability through its manipulation of ABA signaling in order to promote bacterial infection. Since the direct plant target(s) of HopAM1 is unknown, the potential mechanism by which HopAM1 modulates ABA signaling to benefit infection remains unclear.

Although clear evidence demonstrating pathogen factors directly targeting the ABA pathway for virulence is lacking, various pathogenic fungi produce ABA themselves (Dorffling et al. 1984; Jiang et al. 2010). For example, ABA was found in the hyphae of the rice blast pathogen *Magnaporthe grisea* (Jiang et al. 2010) and several other fungal pathogens including *B. cinerea, Fusarium oxysporum* and *Rhizoctonia solani* (Dorffling et al. 1984). Further studies are needed to address the role of the fungi-originated ABA in potential suppression of SA-dependent defense during infection.

### Effectors targeting the growth hormones to indirectly regulate defense

In addition to the “stress” hormone (SA, JA and ET), another group of hormones, including auxin, cytokinin (CK), gibberellin (GA) and brassinosteroid (BR), have been traditionally known as “growth” hormones based on their prominent regulatory role in plant growth and development. Due to extensive crosstalk among hormonal pathways, virulence factors can indirectly suppress plant defense by manipulating the growth hormones. In parallel, pathogens can directly benefit from the modulation of growth hormonal pathways, independent of defense suppression. For example, specific pathogens, such as the gall-forming bacteria, hijack hormonal regulation of plant growth to induce tumorigenesis and acquire shelter and nutrients. Morphological changes due to disturbed homeostasis of growth hormones could also facilitate pathogen dissemination.

### Toxins and effectors suppress plant defense by modulating the auxin pathway

Auxin is the first and most-studied plant hormone that affects almost all aspects of growth and development (Chandler and Werr 2015; Salehin et al. 2015; Schaller et al. 2015). In general, auxin affects plant immunity by acting as a negative regulator (Ludwig-Muller 2015; Naseem et al. 2015). Such an effect is likely achieved through antagonism of auxin signaling on the SA pathway. For example, overexpression of an auxin receptor AUXIN SIGNALING F- BOX PROTEINS 1 (AFB1) led to reduced SA accumulation and enhanced susceptibility during *Pto*DC3000 infection (Robert-Seilaniantz et al. 2011). Furthermore, overexpression of the auxin biosynthetic gene *YUCCA1* (*YUC1*) in SA-deficient plants can further promote infection, suggesting that auxin also impacts defense output in a SA-independent manner (Mutka et al. 2013).

As a negative regulator of defense response, auxin signaling is repressed during infection. For example, perception of bacterial and oomycete PAMPs induces the accumulation of microRNA393 (miR393) in Arabidopsis and soybean, respectively (Navarro et al. 2006; Wong et al. 2014). miR393 contributes to PTI by repressing auxin signaling through its inhibitory effect on the expression of auxin receptors (Navarro et al. 2006). Furthermore, several effectors, such as the bacterial type III effector HopT1-1 produced by *P. syringae* (Navarro et al. 2008) and the oomycete effector PSR1 produced by *P.sojae*, have been shown to suppress the miRNA pathway (Qiao et al. 2013, 2015). These effectors may promote infection by activating auxin signaling and suppressing PTI.

An important strategy to perturb auxin homeostasis is to produce auxin-like molecules, which is quite common in plant pathogens and non-pathogenic microorganisms associating with plants (Manulis et al. 1994; Glickmann et al. 1998; Robert-Seilaniantz et al. 2007). Certain *P. syringae* isolates encode the IAA-conjugation enzyme IAA-LYSINE SYNTHASE (IAAL), which is under the control of a pathogenicity-associated sigma factor. Importantly, deletion of *iaaL* led to reduced virulence, supporting a role of IAA synthesis during *P. syringae* infection (Castillo-Lizardo et al. 2015).

Auxin accumulation and transport can be manipulated via the virulence activities of effectors. The *P. syringae* type III effector AvrRpt2 promotes auxin biosynthesis (Chen et al. 2007) and induces auxin-responsive gene expression by enhancing the proteasomal degradation of AUXIN/INDOLE ACETIC ACID (AUX/IAA) proteins, the key negative regulators of auxin signaling (Cui et al. 2013). Another *P. syringae* type III effector HopM1 associates with ADP ribosylation factor (ARF) guanine nucleotide exchange factor 5 in Arabidopsis (aka AtMIN7) and promotes its degradation. As AtMIN7 is involved in recycling the auxin-efflux carrier PINFORMED (PIN1) (Tanaka et al. 2013), HopM1 may disrupt polar auxin transport and increase plant susceptibility (Nomura et al. 2006). Auxin transport can also be disrupted by the hemibiotrophic oomycete pathogen *Phytophthora parasitica* through the function of PSE1, which affects the expression of the auxin efflux carriers PIN4 and PIN7 (Evangelisti et al. 2013). In this way, PSE1 may facilitate *Phytophthora* infection by disrupting auxin physiology.

Auxin signaling can also be manipulated by viruses. The replicase protein of tobacco mosaic virus (TMV) interacts with the AUX/IAA protein PHYTOCHROME-ASSOCIATED PROTEIN 1 (PAP1) and induces its accumulation in the cytoplasm; consequently, the expression of auxin-responsive genes is repressed due to decreased levels of nuclear-localized PAP1. Importantly, a TMV replicase mutant strain with diminished interaction with PAP1 exhibited reduced viral titer in plants, suggesting that the inhibition of auxin signaling is an important virulence mechanism of TMV (Padmanabhan et al. 2005, 2006, 2008).

### Modulation of the cytokinin pathway to suppress plant defense

Cytokinin regulates defense response in a dosage-dependent manner. Strong activation of CK signaling leads to increased SA levels and confers resistance to biotrophs; on the contrary, subtle or weak activation of CK signaling suppresses PTI, likely in an SA-independent manner (Hann et al. 2014). CK accumulation can be modulated by the type III effector HopQ1, which belongs to a conserved effector family that is produced by a variety of bacterial pathogens including *Pseudomonas* spp., *Xanthomonas* spp. and *Ralstonia* spp. (Hann et al. 2014). HopQ1 possesses a hydrolase activity that can catalyze the conversion of CK precursors to active forms. As a result, HopQ1 expression in Arabidopsis led to elevated accumulation of CK and hypersusceptibility to bacterial infection. Interestingly, exogenous applications of low doses of CK repressed the transcription of the PAMP-receptor gene *FLAGELLIN SENSING 2* (*FLS2*), indicating that HopQ1 dampens PTI by suppressing PRR expression (Hann et al. 2014).

### Effectors that potentially manipulate the brassinosteroid pathway

As a relatively young hormone, brassinosteroid (BR) has been shown to regulate a wide array of developmental processes and stress responses (Kong et al. 2012; Zhu et al. 2013; De Bruyne et al. 2014). Indeed, BR signaling has been reported to regulate plant defense both positively (Chinchilla et al. 2007; Heese et al. 2007) and negatively (De Vleesschauwer et al. 2012; Nahar et al. 2013). Current studies of BR-mediated regulation in defense are centered on the BRI1 ASSOCIATED RECEPTOR KINASE 1 (BAK1/SERK3). Originally identified as a co-receptor of the BR receptor BRASSINOSTEROID INSENSITIVE 1 (BRI1) (Li et al. 2002; Nam and Li 2002), BAK1 was later discovered to associate with multiple PRRs and play an essential role in ligand binding and PTI signaling (Chinchilla et al. 2007; Heese et al. 2007; Sun et al. 2013). Remarkably, several bacteria effectors directly target BAK1 and suppress PTI. The first examples of BAK1-manipulating type III effectors are AvrPto and AvrPtoB of *P. syringae* (Shan et al. 2008; Cheng et al. 2011). By suppressing the kinase activity of BAK1 and PRRs (Shan et al. 2008; Xiang et al. 2008; Cheng et al. 2011), AvrPto and AvrPtoB play an essential role in bacterial infection, primarily by disrupting PTI signaling. Two additional type III effectors, HopF2 produced by *P. syringae* and Xoo2875 produced by the rice pathogen *Xanthomonas oryzae* pv. *oryzae* (*Xoo*), can also associate with BAK1 in Arabidopsis and rice respectively (Yamaguchi et al. 2013; Zhou et al. 2014); however, how HopF2 and Xoo2875 may manipulate BAK1 function remains to be elucidated.

A challenge to dissect the contribution of BR to defense response is the dual roles of BAK1 in both PTI and BR signaling (Albrecht et al. 2012; Belkhadir et al. 2012). In addition, BAK1 has also been shown to act as a co-receptor for PEP RECEPTOR1 (PEPR1), which is involved in the perception of damage-associated molecular patterns (DAMPs) (Schulze et al. 2010). Provided the diverse roles of BAK1 in distinctive signaling pathways, whether these BAK1-targeting effectors benefit infection through a potential disruption of BR signaling, as well as other pathways such as the DAMP signaling, requires careful investigation. Furthermore, BR signaling has been proposed to negatively regulate immunity through a role in regulating growth/defense tradeoff (Lozano-Duran and Zipfel 2015). Therefore, it is possible that pathogens may benefit from manipulating BR signaling through targeting components not directly involved in PTI.

### Pathogen hijacking growth hormone homeostasis to enhance nutrient acquisition

**Fig. 2:**
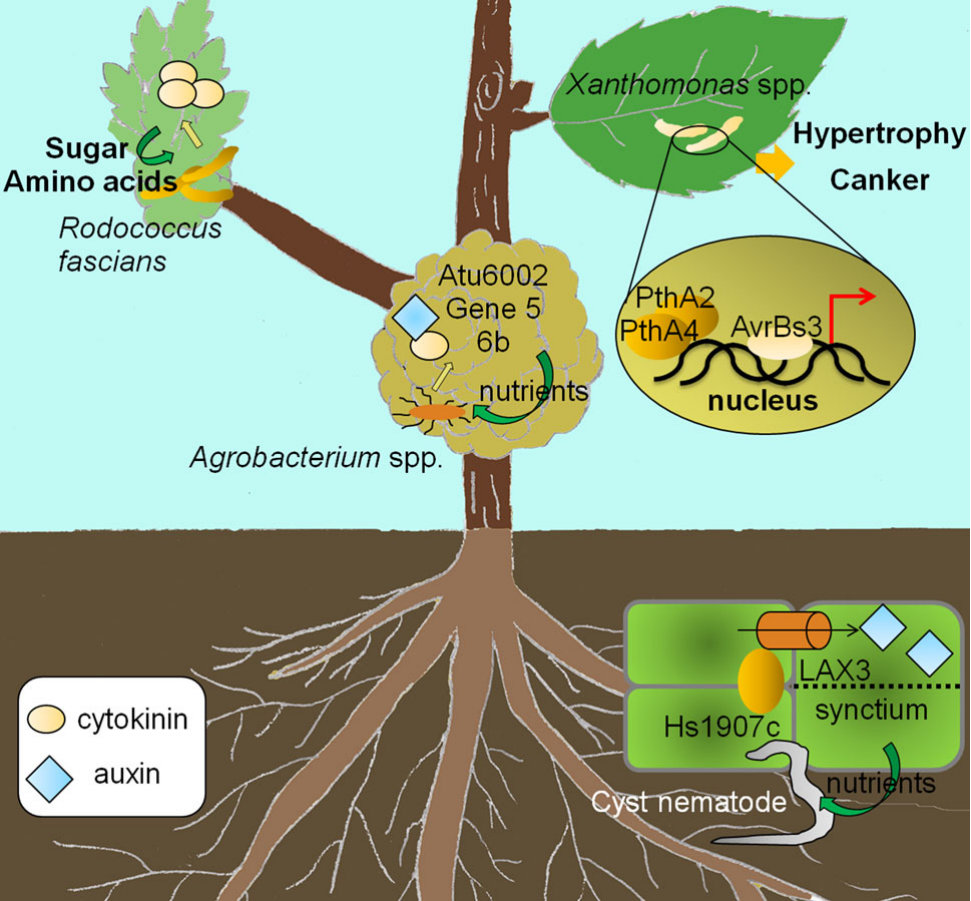
Effectors interfering with growth hormones cytokinin and auxin to acquire shelter and nutrients, as well as to facilitate pathogen dissemination

In addition to defense suppression, pathogens gain additional benefits from disturbing growth hormone homeostasis (Fig. 2). One of the benefits is nutrient allocation into infected tissues for sustained pathogen proliferation. Many pathogens produce cytokinins (CKs) to facilitate translocation of nutrients into infected sites. These areas are known as “green islands” because they exhibit delayed senescence and support continuous growth of the pathogen population (Walters and McRoberts 2006; Walters et al. 2008).

Gall-forming bacteria are well-known for their ability to produce CK. For example, the bacterial pathogen *Rhodococcus fascians* produces a mixture of six different CK mimics. Genes involved in CK biosynthesis and metabolism are encoded on a large linear plasmid pFiD188, and the loss of pFiD188 rendered the bacterium unable to cause disease (Crespi et al. 1992; Radhika et al. 2015). However, introduction of one CK biosynthesis gene cluster into this avirulent mutant failed to complement the phenotype (Crespi et al. 1992). Therefore, it is likely that various forms of CKs produced by *R. fascians* work together to facilitate infection (Depuydt et al. 2009a; Pertry et al. 2009, 2010). Interestingly, high levels of sugar and amino acids are accumulated in *R. fascians*-induced galls; these nutrients are presumably resulted from the production of CKs by *R. fascians* to support bacterial proliferation (Depuydt et al. 2009b).

The crown gall disease pathogens *Agrobacterium* spp. carry auxin and CK biosynthetic genes within the T-DNA region, which are incorporated into the genome of susceptible plants to induce tumorigenesis (Schroder et al. 1984; Thomashow et al. 1986). Moreover, additional *Agrobacteria* proteins contribute to gall formation by fine-tuning hormonal flux and the ratio between auxin and CK. For examples, gene 5 acts as an auxin antagonist and regulates auxin responsiveness through an autoregulatory loop (Korber et al. 1991); Atu6002 interferes with the perception of auxin in tumor-forming cells (Lacroix et al. 2014); and 6b affects the localization of auxin, and possibly CK, in tumor cells (Takahashi et al. 2013). It is proposed that these proteins function together to promote shoot formation on galls, which extends the lifespan of infected hosts and safeguards a stable food source for the bacterium.

Plant pathogenic nematodes are also known to produce effectors, which can promote infection through the manipulation of the hormonal pathways (Mitchum et al. 2013). Cyst nematodes induce the formation of specialized feeding structures known as syncytia in plant roots. Syncytia are formed by fusions of neighboring root cells and serve as nutrient sinks for the nematodes. Beet cyst nematode produces an effector Hs1907C, which interacts with the AUXIN INFLUX CARRIER (LAX3) and potentially directs the influx of auxin into root cells (Lee et al. 2011). Auxin can then induce lateral root formation and the production of cell wall-modifying enzymes (Swarup et al. 2008), which facilitate syncytia development. Interestingly, transgenic plants expressing Hs1907C are more resistant to cyst nematodes. A possible explanation is that the overall disturbance of auxin flux in roots may reduce the amount of auxin available for the nematode-feeding sites (Swarup et al. 2008; Lee et al. 2011). If this is true, spatial expression of effector disrupting hormonal homeostasis would be pivotal in determining the outcome of infection.

### Effectors manipulate growth hormones to promote dissemination

The last step to complete an infection cycle is the dissemination of pathogens to new hosts. Several type III effectors known as TRANSCRIPTION ACTIVATOR-LIKE effectors (TALEs) are produced by *Xanthomonas* spp. to promote bacterial dissemination by directly activating growth hormone-responsive genes in plant hosts. AvrBs3 from *X. campestris* activates the expression of the transcription factor Upa20 in pepper that regulates auxin-responsive genes. Among them, an expansin-like gene induces hypertrophy of mesophyll cells, which may enhance bacterial release from lesions (Marois et al. 2002; Kay et al. 2007). Similarly, two TALEs, PthA2 and PthA4, produced by the citrus canker pathogen *X. citri* were shown to induce genes involved in ET, GA and auxin signaling pathways. Even though the biological significance of the elevated expression of these genes requires further investigation, gene ontology enrichment analysis suggested that these genes may be associated with cell wall modifications (Pereira et al. 2014), which promote the development of canker pustules (Cernadas and Benedetti 2009). Therefore, PthA2 and PthA4 may contribute to canker formation and facilitate bacterial dissemination to infect new hosts.

## Conclusions and future perspectives

Constantly challenged by potential pathogens in the environment, failure to mount an effective defense response is fatal to plants. Plant hormones have profound impact on immunity, not only through the canonical “defense” hormones, but also through the “growth” hormones, which exhibit bifurcated functions in modulating defense as well as regulating resource allocation (Huot et al. 2014). During the co-evolutionary arms race with their hosts, pathogens have evolved sophisticated strategies to maximize fitness in plants. Therefore, it is not surprising that the plant hormone network has been repeatedly identified as targets of a broad range of pathogens. Although most studies have focused on defense suppression, hijacking the nutrient allocation system and disturbing growth-defense tradeoff is of critical importance to pathogenesis. Therefore, pathogen manipulation of growth hormonal signaling for benefits independent of suppressing immunity cannot be overlooked.

Even though the distinctive roles of SA and JA in regulating defense to biotrophs and necrotrophs have generally been accepted, contradictory findings were also reported. Such discrepancies may be attributed to the use of transgenic plants or hormone analogs in simplified laboratory studies. It is likely that endogenous hormone levels during natural infections hardly fluctuate to a level resulted from experimental manipulations. Since hormonal regulation is a dynamic process with intricate crosstalk, cautions need to be taken when interpreting data from experiments involving extensive perturbation of the network. Changes in nutrient requirement and virulence strategies at different infection stages should also be considered as determining factors to understand specific manipulation of hormonal pathways by a particular pathogen.

Finally, many plant-associating microorganisms including pathogens and symbionts are shown to directly produce plant hormones. Currently, it is not well understood how these additional hormonal signals generated from the phytobiome may impact plant physiology and pathogen infection. Furthermore, symbiotic organisms can also produce effectors to modulate plant hormonal network and foster colonization (Plett et al. 2014). Studies of effectors from symbiotic microbes and the roles of microbe-originated hormones will provide new insight into our understanding of plant-microbe interactions.
